# Ataulpho de Paiva Foundation on the stage of BCG

**DOI:** 10.1590/S1677-5538.IBJU.2021.03.02

**Published:** 2021-03-25

**Affiliations:** 1 Fundação Ataulpho de Paiva Rio de JaneiroRJ Brasil Fundação Ataulpho de Paiva, Rio de Janeiro, RJ, Brasil.

## COMMENT

The Ataulpho de Paiva Foundation (FAP) is a private non-profitable institution, with around 180 employees, founded on August 4, 1900 as the Brazilian League Against Tuberculosis. In 1930, FAP started to produce and vaccinate, free of charge, the population of Rio de Janeiro with the BCG Moreau Rio de Janeiro Tuberculosis vaccine. From then on, the vaccine was distributed by FAP to Institutes in other Brazilian States.

The BCG Moreau Rio de Janeiro strain is owned by FAP and has its own characteristics causing fewer adverse effects, greater immunogenicity and is one of the most studied BCGs in the world. The Moreau RJ strain is certified by WHO and the vaccine is also a WHO vaccine reference. The largest clinical study done with BCGs in the world was carried out in Brazil, with our vaccine, and showed that Brazilian BCG causes few adverse effects and that they are mild (1). Brazilian health authorities have immunized more than 150 million people with our vaccine, thus being the most used vaccinein the country with coverage above 90% of the Brazilian population.

One of the products that FAP produces is ImunoBCG which is the treatment for Superficial Bladder Cancer, this product has been on the market since 2005, based on research that we conducted with FINEP. It was initially manufactured only at our São Cristovão factory. Which was closed by Anvisa until we adapted to the new standards of Good Manufacturing Practices (GMP). From our reopening in early 2020, after meeting all Anvisa's requests, we manufactured the product in its initial phase (biological and pharmaceutical) at the renovated São Cristovão factory and the final phase (packaging, storage and distribution) has been carried out in the recently opened GMP Xerém factory. Since then, around 120,000 doses of ImunoBCG have been manufactured in 2020, which has supplied the market.

We are currently transferring the manufacturing technology of ImunoBCG to the Spanish company Biofabri (Zendal), which is carrying out a large clinical study in several Spanish Urological Centers. So FAP is making one of the first technology transfers of a Brazilian immunobiological product to Europe (https://clinicaltrials.gov/ct2/show/NCT03982797).

The FAP Institution, through its social activity, provides basic social protection services to children from 2 to 12 years of age in situations of vulnerability and social risk, in its unit on the Island of Paquetá, Rio de Janeiro. In the city of Duque de Caxias (RJ), we've carried out a program of active search for tuberculosis cases and reduction of treatment abandonment. Fifteen health agents work in the area, visiting households, in an area with around 50,000 inhabitants, tracing the social and health profile of the inhabitants, providing guidance, counseling and monitoring the treatment and evolution of the occurrences of tuberculosis and other diseases - such as AIDS and leprosy.
